# Rabbit-based experimental model for scalp reimplantation: advancing microsurgical training and translational research

**DOI:** 10.1590/acb403725

**Published:** 2025-04-28

**Authors:** Rui Sergio Monteiro de Barros, José Maciel Caldas dos Reis, Deivid Ramos dos Santos, Vitor Nagai Yamaki, Renan Kleber Costa Teixeira, André Lopes Valente

**Affiliations:** 1 Porto Dias Hospital – Department of Orthopedics and Traumatology – Belém (PA) – Brazil.; 2 Porto Dias Hospital – Department of Orthopedics and Traumatology – Belém (PA) – Brazil.; 3 Universidade do Estado do Pará – Postgraduate Program in Surgery and Experimental Research – Belém (PA) – Brazil.; 4 Universidade de São Paulo – Faculdade de Medicina de São Paulo – Hospital das Clínicas – Department of Neurosurgery – São Paulo (SP) – Brazil.; 5 Universidade do Estado do Pará – Postgraduate Program in Surgery and Experimental Research – Belém (PA) – Brazil.; 6 Universidade do Estado do Pará – Postgraduate Program in Surgery and Experimental Research – Belém (PA) – Brazil.

**Keywords:** Microsurgery, Education, Medical, Rabbits, Scalp

## Abstract

**Purpose:**

To develop an experimental model of microsurgical scalp reimplantation in rabbits.

**Methods:**

Ten male albino New Zealand rabbits (Oryctolagus cuniculus) were scalped and subjected to scalp reimplantation. The rabbits’ scalp regions, including their ears, were surgically amputated. Based on a previous anatomical study, the superficial temporal artery and the central auricular vein were chosen for microanastomosis. Data on the morphometric parameters (vessel weight and caliber), surgical procedure (surgical time and number of stitches), and surgical recovery parameters (venous return, patency, and necrosis) were collected for up to 30 days postoperatively (PO) using a research protocol.

**Results:**

Morphometric and surgical recovery parameters did not significantly differ in our sample. No animals died during the intraoperative period. Three animals were euthanized because they developed partial or total scalp necrosis. The venous return was impaired from three to ten days PO, with spontaneous regression after this period, which significantly improved (*p* = 0.02) after 14 days PO. Superficial necrosis was observed starting at two days PO with complete resolution by day 21 PO (*p* <0.01).

**Conclusion:**

The rabbit provides a realistic biological model for training scalp reimplantation with high fidelity to human vascular structures.

## Introduction

The scalp consists of five distinct layers, described by the SCALP acronym (Skin, Connective tissue, Aponeurosis, Loose areolar connective tissue, and Pericranium). These layers provide protection to the calvarium and support vital structures. The term *scalping*, widely used in media, refers to the partial or complete traumatic removal of the scalp, a rare but devastating injury^1^. The term scalping commonly used in the media indicates the partial or total traumatic removal of the scalp^2^. Total scalp removal is rare in the literature but is devastating^3^, causing victims to experience severe physical problems and intense psychological and social suffering with grave consequences for their self-esteem, bodily perception, and finances^4^.

Although common in many parts of the world during the early stages of industrialization, scalping still occurs in developing countries due to the misuse of industrial equipment or use it without proper personal protective equipment^3^^,^ 4, as it happens in the Amazon region, with the registration of 112 cases in the state of Pará (16 cases only in 2021)^5^^,^ 6, Brazil, disagreeing other literature reports^7^^,^ 8.

In Amazonia, scalping usually involves the riverine population, whose primary mode of transport are rivers. In most cases, injuries occur in women whose hair is accidentally trapped in exposed boat motor axes^4^^,^ 5. Scalping can be accompanied by other lesions, including traumatic brain injury and trauma to the spinal cord; it may even lead to death^9^^,^ 10.

Public health centers specialized in reimplantation are unavailable in the Amazonian region. The restorative surgical resources available to treat scalping are limited to the trepanation of exposed cranial bones followed by an extended period of granulation tissue development and tissue coverage using skin grafts^6^. This procedure is aesthetically damaging to these women who often use wigs to hide the deformation.

Therefore, despite the relatively high scalping rate in the Amazon, managing this condition remains unaddressed in the literature. New studies are needed to provide more reliable incidence records, a detailed epidemiological population profile, and better health care for victims of this condition.

Many studies involving scalp reimplantation have indicated better aesthetic and functional results versus repair surgery^11^_–_ 14. In the field of animal experimentation, rabbits are used because of their docile aspect, easy manipulation, besides their great similarity to human anatomy and physiology and studies showing vascular anastomoses with good results^15^^,^ 16.

Despite the great importance of the rabbit for animal experimentation, no studies were found that approached its use as a model in microsurgical scalp repair. This shows a still little explored field and scarce information on this theme that may subsidize research on leather reimplantation treatments.

Considering the high rate of scalping cases and the scarcity of microsurgical resources in the state of Pará, this study developed an experimental model of scalp reimplantation based on a previous vascular anatomical study to assess the possibility of disseminating this model.

## Methods

### Ethics and animals use

This study was approved by the Animal Research Ethics Committee of the Universidade do Estado do Pará (UEPA) under protocol no. 34/2024. All procedures complied with the current legislation for the use of experimental animals (Federal Law No. 11.794 of October 8, 2008) and adhered to the ethical principles of the Brazilian College of Animal Experimentation. Measures were taken to minimize animal suffering, including the use of appropriate anesthesia, analgesia, and postoperative care.

Rabbits were selected as the experimental model due to their anatomical size and physiological characteristics, which provide a realistic platform for surgical training. Their larger anatomy allows for procedures that replicate real-world challenges, particularly in pediatric scalp avulsion cases–a frequent scenario in the studied context. This anatomical fidelity is crucial for preparing surgeons to handle complex microsurgical interventions with precision and confidence.

This study extends beyond basic microvascular anastomosis training, encompassing the entire surgical process, from avulsion to reimplantation and postoperative evaluation. Rabbits offer physiological conditions suitable for replicating this comprehensive surgical sequence. Their vascular structures, documented for their similarity to human microcirculatory systems, and their docile and manageable nature make them an ideal model. These attributes address regional demands for effective microsurgical training and contribute to advancing clinical outcomes in reconstructive procedures.

#### Animal care and handling

Ten male albino New Zealand rabbits (*Oryctolagus cuniculus*), aged 6 to 12 months and weighing 2 to 3 kg, were reared at the Central Animal Facility of the Universidade Federal Rural da Amazônia. Before surgery, animals underwent a 15-day adaptation period at the Animal Facility of the Laboratory of Experimental Surgery, at UEPA. Environmental conditions were maintained at 22°C with a relative humidity of approximately 60% and a 12:12-hour light-dark cycle. Feed and water were provided *ad libitum*, and animals were housed individually in cages sanitized three times weekly to ensure optimal hygiene and comfort.

#### Study type

The study was longitudinal, experimental, and interventional.

#### Surgical procedure

The animals were subcutaneously anesthetized with ketamine (70 mg/kg) and xylazine (10 mg/kg). When necessary, half the starting dose was administered to maintain anesthesia. After achieving the anesthetic plane, enoxaparin (anticoagulant, 1 mg/kg) was applied subcutaneously; 10% enrofloxacin (antibiotic, 15 mg/kg) and tramadol (analgesic, 1 mg/kg) were then administered intramuscularly. These drugs were administered once daily for seven days postoperatively (PO).

Before beginning the surgical procedure, the hair was trichotomized in the region to be incised, and the skin was cleaned and disinfected. All animals were subjected to the same surgical protocol. A surgical microscope was used (D. F. Vasconcelos, Brazil) with a magnification of up to 40×.

**Figure 1:**
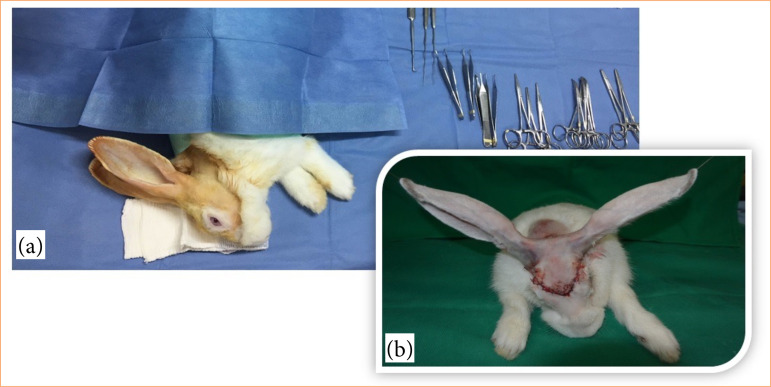
Preoperative preparation and immediate postoperative aspect in a surgical experimental model. **(a)** Preoperative preparation of the animal. **(b)** Immediate postoperative period.

Source: Elaborated by the authors.

The surgical access route was standardized by simulating total removal of the scalp in the junction line between the scalp, face, and cervical region in which the skin adherence was weaker in addition to other structures that may be removed during the total scalp avulsion, including the ears (Fig. 1).

Both the rabbit’s ears were surgically detached along with the adjacent skin (scalp) with a 2-cm radius from the ear base relative to the midline. The surgical incision limits were anterior to the supraorbital region, until the base of the muzzle and lateral and posterior by a circumferential incision with a 2-cm radius around the ear base on both sides.

The sequence of scalping and vessel flushing involved targeted administration rather than systemic dosing. Initially, after partial dissection of the vessels but before completing the scalping, 200 mL of Ringer’s lactate (RL), 5,000 IU of heparin, and 20 mL of 2% lidocaine were used to flush the vessels locally. This procedure was aimed at preventing clot formation and ensuring adequate vessel preparation for subsequent anastomosis. Care was taken to avoid systemic absorption by confining the flushing solution strictly to the vessel lumen. After this step, the scalping procedure was finalized.

The superficial temporal artery was initially approached at each ear base followed by the central auricular vein. Blood flow was temporarily interrupted in these vessels with a double microclamp to finish scalping.

**Figure 2:**
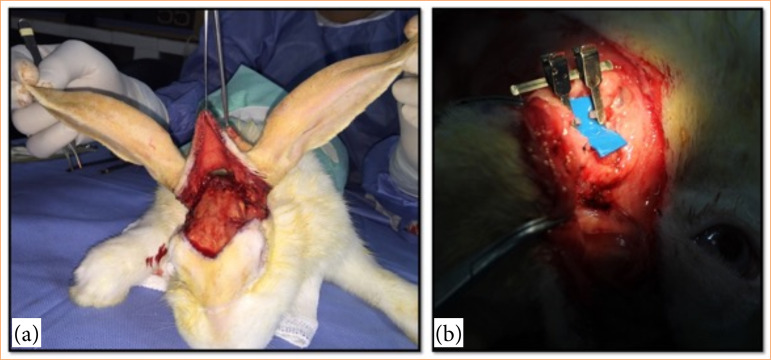
Demonstration of the intraoperative phase. **(a)** Scalp avulsion leaving only the vascular pedicle intact. **(b)** The superficial temporal artery was dissected and transected.

Source: Elaborated by the authors.

The scalp was then microsurgically re-implanted. A small piece of blue plastic was used in the blood vessels for contrast (Fig. 2). The sectioned vessels were carefully dilated and later anastomosed with single stitches using 10-0 mononylon thread and a BV75-4 needle (Ethilon, Edinburgh, Scotland). Blood vessel patency was evaluated using the “milking test”8. The procedure was considered successful when the vessel filled with blood after milking indicating adequate blood flow.

The scalp was then reattached by suturing the external auditory canal with 4-0 nylon and suturing the skin with 5-0 nylon. The animals were transferred to appropriate cages after surgery.

#### Evaluated parameters

The morphometric parameters analyzed were the weight (kilograms) and caliber of the arterial and venous vessels approached (millimeters).

The following surgical parameters were evaluated: total surgical time (min) from the first incision to the end of the last stitch on the animal’s skin, arterial and venous anastomosis time from the first stitch passage to declamping, number of stitches in the anastomoses, and the vascular patency during the intraoperative period as measured using the milking test^14^.

**Figure 3:**
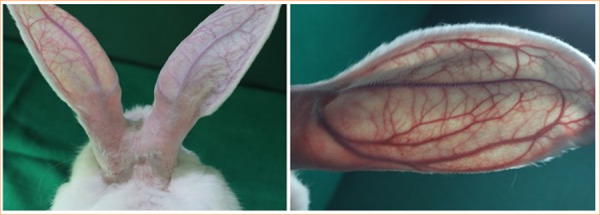
The circulation of the rabbit’s ear was evaluated using transillumination.

Source: Elaborated by the authors.

The following external parameters were analyzed by daily clinical evaluation for 30 days PO: hair emergence, venous return quality, scalp necrosis, and vascular patency in the ear using transillumination and the milking test (Fig. 3).

#### Statistical analysis

The data were organized into databases, and tables and graphs were created using Microsoft Excel and Word. Data were analyzed using BioEstat version 5.4. One-way analysis of variance (ANOVA) and Student’s t-test were used for numerical variables; Fisher’s exact test was used for categorical variables and multivariate analyses to compare animal survival. The value of *p* < 0.05 was used to reject the null hypothesis.

### Results

**Figure 4:**
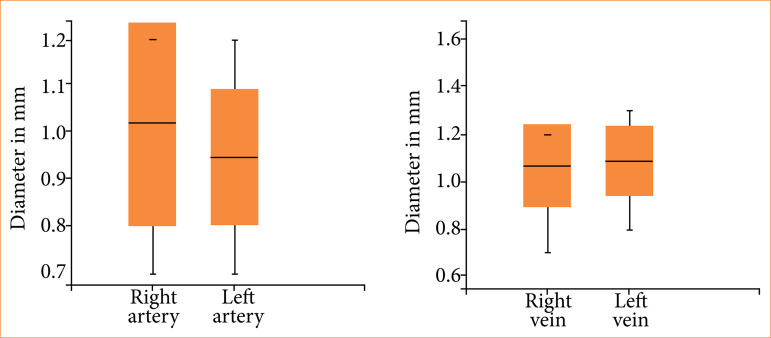
Mean diameters of the arteries and veins in the rabbit scalps. Right artery *versus* left artery (*p* = 0.40), right vein *versus* left vein (*p* = 0.78), arteries *versus* veins (*p* = 0.27).

The animals’ mean weight was 2.75 ± 0.50 kg. The arterial diameters ranged from 0.7 to 1.2 mm with no significant differences (*p* = 0.40) between the right and left sides. The venous diameters ranged from 0.7 to 1.3 mm with no significant differences (*p* = 0.78) between the right and left sides (Fig. 4). Animal weight and vessel diameter were not significantly correlated (*p* > 0.05).

*Student’s t-test and analysis of variance. Source: Elaborated by the authors.

**Figure 5:**
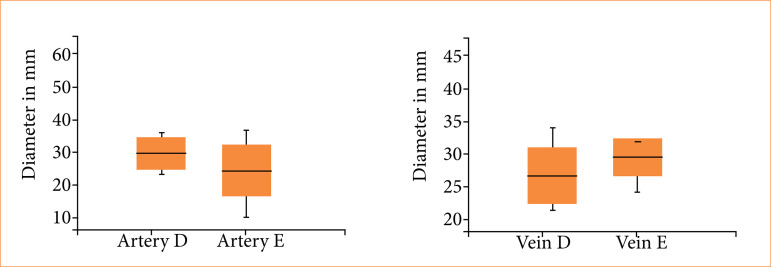
Mean time of anastomosis (in min) of the arteries and veins in the rabbit scalps. Right artery *versus* left artery (*p* = 0.07), right vein *versus* left vein (*p* = 0.66), arteries *versus* veins (*p* = 0.16).

The surgical time varied from 245 to 330 min with a mean of 290 ± 28 min. The arterial anastomosis time ranged from 11 to 36 min with no significant differences (*p* = 0.07) between the right and left sides (29.9 ± 4.62 and 24.7 ± 7.40, respectively) (Fig. 5). The venous anastomosis time varied from 22 to 44 min with no significant differences (*p* = 0.66) between the right and left sides (28.6 ± 6.67 and 29.6 ± 2.75, respectively).

*Student’s t-test and analysis of variance. Source: Elaborated by the authors.

The number of stitches in the arteries and veins ranged from six to nine, with a mean of 7.20 ± 0.69 in the arteries and 6.80 ± 1.00 in the veins with no significant differences (*p* = 0.99) between the right and left sides. Patency in the arteries and veins was 100% during the intraoperative period.

No animals died during the intraoperative period. Three animals were euthanized due to partial or total scalp necrosis (rabbit 1 on day 10 PO and rabbits 3 and seven PO).

In the multivariate analysis, the weight (*p* = 0.83), arterial diameter (*p* = 0.76), venous diameter (*p* = 0.81), arterial anastomosis time (*p* = 0.88), venous anastomosis time (*p* = 0.79), and number of stitches (*p* = 0.75) did not significantly affect animal survival.

**Figure 6:**
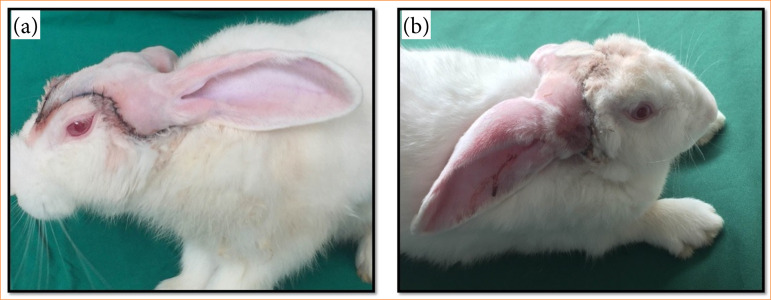
Demonstration of the postoperative phase. **(a)** Postoperative day 3; **(b)** postoperative day 7.

Source: Elaborated by the authors.

**Figure 7:**
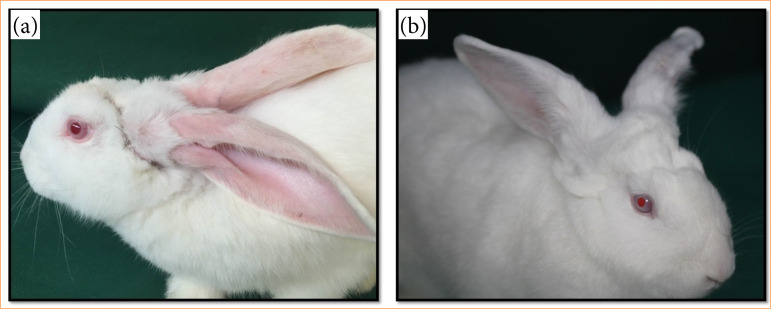
Demonstration of the postoperative phase. **(a)** Postoperatively day 14; **(b)** postoperative day 30.

Source: Elaborated by the authors.

External clinical parameters revealed that hair regrowth began at three days PO, with all animals exhibiting complete fur regrowth by 14 days PO (*p* = 0.04). Impaired venous return was observed between days 3 and 10 PO, with spontaneous recovery occurring afterward and significant improvement by day 14 PO (*p* = 0.02). Superficial necrosis of the ears and scalp was noted from day 2 PO and resolved completely without intervention by day 21 PO (*p* < 0.01). These findings demonstrate the progressive postoperative recovery of the animals, as illustrated in Figs. 6 and 7.

## Discussion

Although rare in clinical practice, scalp injuries present significant challenges to reconstructive surgery due to their complexity^4^. While reimplantation offers superior aesthetic and functional outcomes compared to other techniques, the rarity of these cases limits opportunities for routine surgical training. This study addresses this gap by introducing a rabbit-based experimental model of scalp reimplantation, providing a reproducible platform to advance microsurgical training and promote translational research^6^^,^ 8 ^,^ 9 ^,^ 16–19.

Rabbits were selected for this study due to their favorable anatomical and vascular characteristics, including vessel diameters that closely mimic human microcirculatory systems. Rabbits were utilized as the experimental model in this study due to their anatomical and physiological features, which closely resemble human vascular systems and offer a practical framework for surgical training. Their larger body size facilitates the simulation of realistic surgical challenges, particularly relevant for pediatric scalp avulsions–a common scenario in this context–providing a high degree of anatomical similarity essential for training in advanced microsurgical techniques.

This study goes beyond isolated microvascular anastomosis practice by encompassing the entire surgical process, from the initial avulsion to reimplantation and subsequent postoperative evaluation. The suitability of rabbits for replicating this complex sequence, combined with their well-documented similarities to human vascular structures and ease of handling, underscores their effectiveness as a model for refining surgical expertise, addressing local training needs, and enhancing outcomes in complex reconstructive procedures. Buncke and Schulz^14^ used isolated reimplantation of rabbit ears as a training model for digital reimplantation with good results.

The blood vessel weight and caliber were consistent across the sample, with minimal variation and no statistically significant differences. Similarly, the number of stitches required for effective arterial and venous anastomoses showed uniformity among animals, confirming the suitability of rabbits as experimental models. A thorough review of major scientific databases revealed no prior studies on experimental scalp reimplantation, highlighting the novelty of our findings and the lack of comparative data in the existing literature.

An anatomical study of the scalp and ear vascularization in rabbits may help identify essential characteristics of the vascularization of the arteries and veins and their main branches. Therefore, the scalp area standardized for amputation and the surgical approach for reimplantation were chosen based on the blood flow provided by the superficial temporal artery and central auricular vein.

The comparable arterial and venous anastomosis times may be explained by the technical challenges of arterial anastomosis, which is positioned deeper within the surgical field compared to the more accessible subcutaneous location of the central auricular vein.

The literature describes a high success rate for microsurgical scalp reimplantation in humans with the necrotic areas varying widely^7^^,^ 18 ^,^ 20 ^,^ 21. In this study, two animals (20%) experienced necrosis affecting approximately 50% of the re-implanted scalp, while one animal developed an infection that resulted in complete necrosis.

Three animals were euthanized due to partial or total scalp necrosis, likely resulting from the complexity of the procedure and challenges in maintaining clinical parameters during surgery. To preserve methodological consistency, no additional reconstructive interventions were performed on the animals. However, in clinical practice, various techniques are utilized to address tissue loss, including serial debridement, skin grafting, cutaneous and muscular flaps, and surgical revisions^3^^,^ 7 ^,^ 21_–_ 24.

Nasir et al.^22^ found that the mean surgical time ranged from 5 to 10 h. The mean surgical time for our sample was 290 min (4.8 h) under the experimental conditions used.

Multivariate analysis reported no significant morphometric or surgical parameters, suggesting that partial or total loss of the graft was not due to the proposed technique but possibly due to other factors unevaluated in this study, including clinical complications or external factors.

The prognosis for total scalping is usually better than that for partial scalping, because total scalping involves more vessels that are more easily identified; the vessel calibers are more favorable for reconstruction^25^^,^ 26. Although previous studies indicated that a single artery could provide blood supply to the entire scalp during reimplantation in humans^23^, venous drainage is a cause for concern because scalp losses are primarily due to failed venous drainage^3^^,^ 19 ^,^ 20 ^,^ 21 ^,^ 27. Yin et al.^21^ reported that the only flaw in the presented series was the use of a single arterial source without venous drainage. This approach may have favored venous congestion that evolved to necrosis. This outcome has changed surgeons’ behavior for maintaining the best possible venous drainage with multiple venous anastomoses and grafts^9^^,^ 19 ^,^ 21 ^,^ 23 ^,^ 28.

Hemiscalp necrosis was observed in two rabbits, but it did not spread to the contralateral side suggesting vascular independence between the two systems. This situation differs from the human scalp, whose vascularization allows anastomosis of only one vascular pedicle for supplying blood to the entire scalp (one artery and one vein)^3^^,^ 23. This result corroborated the study of Buncke and Schulz^14^, in which the ears were independently re-implanted, and the necrosis levels differed between the right and left sides. Thus, creating a scalping model for the hemiscalp may be possible.

Regarding external clinical parameters, hair emergence is the earliest sign of the graft’s good vitality. New fur growth suggested that blood flow was adequately nourishing the scalp. Adverse events from the procedure (poor venous return and superficial necrosis) demonstrated the need for a follow-up of at least 25 days to completely restore the animal’s physiology (regression of scalp and ear edema and spontaneous resolution of superficial necrosis).

Poor venous return, which was clinically translated as venous congestion of the scalp and ears, was common and expected in the first days after surgery; all animals presented this complication until three days PO. Nonetheless, this complication resolved spontaneously and began to regress 15 days PO for all animals. In contrast, maintaining venous congestion after this period indicates a possible limitation of follow-up and progression to obvious hemiscalp necrosis. Superficial necrosis was common in our sample particularly around the stitches and on the ear bases. However, necrosis without intervention resolved spontaneously leading to healthy tissue formation.

The venous impairment observed in our model, followed by spontaneous improvement attributed to random revascularization along the wound edges, represents a significant limitation in evaluating venous drainage success in reimplantation. To enhance its applicability, we propose modifications that include performing additional venous anastomoses and using vascular grafts to ensure more consistent and controlled venous drainage. These improvements would not only strengthen the model’s reproducibility but also increase its translational relevance for clinical studies, reducing reliance on spontaneous processes and optimizing experimental outcomes.

One advantage of the proposed model was the possibility of evaluating the blood flow during the postoperative period using transillumination. This technique allows one to noninvasively evaluate blood flow without requiring anesthesia. Analysis of this technique indicates that all analyzed vessels had adequate blood flow in the main artery and marginal veins of the ears. In contrast, arterial and venous blood flow could not be identified using this technique in the animals with hemiscalp necrosis during the two days preceding complete necrosis.

We did not perform traumatic scalp amputation in rabbits because of the ethical limitations on mechanically removing the scalp and the difficulty in standardizing the injuries. Other study limitations were that scalping was performed surgically, and the scalps were re-implanted immediately. These features contrast with traumatic scalping in humans that involves greater lesion complexity, prolonged ischemia, and injuries to other systems^12^^,^ 19 ^,^ 23_–_ 28. However, immediate surgical reimplantation reduced the possible effect of reperfusion injury on the repaired tissues’ viability.

This model constitutes the best surgical approach for traumatic scalp injuries and reimplantation. Therefore, this study may offer continued experimental microsurgical training for teams of professionals. Effective scalp treatments are possible in Brazil using translational research29 from experimental studies in clinical practice; this minimizes the physical and psychological sequelae.

## Conclusion

Rabbits serve as a highly realistic biological model for scalp reimplantation training, offering anatomical and vascular structures closely resembling those of humans. Its larger size and physiological characteristics provide a practical platform not only for mastering microsurgical techniques but also for replicating the entire surgical process, from avulsion to reimplantation and postoperative care. Consequently, rabbits represent a reliable and translational experimental model for advancing research and training in complex microsurgical procedures, particularly in addressing the demands of clinical practice and surgical education.

## Data Availability

The data will be available upon request
